# Nanoparticles of Bioactive Glass Enhance Biodentine Bioactivity on Dental Pulp Stem Cells

**DOI:** 10.3390/ma14102684

**Published:** 2021-05-20

**Authors:** Camila Corral Nunez, Diego Altamirano Gaete, Miguel Maureira, Javier Martin, Cristian Covarrubias

**Affiliations:** 1 Department of Restorative Dentistry, Faculty of Dentistry, Universidad de Chile, Santiago 8380544, Chile; dgaete1892@gmail.com (D.A.G.); jamartin@odontologia.uchile.cl (J.M.); 2 Laboratory of Nanobiomaterials, Research Institute of Dental Sciences, Faculty of Dentistry, University of Chile, Santiago 8380544, Chile; miguel.maureira@bq.uchile.cl

**Keywords:** apatite-forming ability, bioactive glass, bioactivity, nanocomposites

## Abstract

This study aimed to investigate the cytotoxicity and bioactivity of a novel nanocomposite containing nanoparticles of bioactive glass (nBGs) on human dental pulp stem cells (hDPSCs). nBGs were synthesized by the sol–gel method. Biodentine (BD) nanocomposites (nBG/BD) were prepared with 2 and 5% wt of nBG content; unmodified BD and glass ionomer cement were used as references. Cell viability and attachment were evaluated after 3, 7 and 14 days. Odontogenic differentiation was assessed with alkaline phosphatase (ALP) activity after 7 and 14 days of exposure. Cells successfully adhered and proliferated on nBG/BD nanocomposites, cell viability of nanocomposites was comparable with unmodified BD and higher than GIC. nBG/BD nanocomposites were, particularly, more active to promote odontogenic differentiation, expressed as higher ALP activity of hDPSCs after 7 days of exposure, than neat BD or GIC. This novel nanocomposite biomaterial, nBG/BD, allowed hDPSC attachment and proliferation and increased the expression of ALP, upregulated in mineral-producing cells. These findings open opportunities to use nBG/BD in vital pulp therapies.

## 1. Introduction

Current scientific evidence has provided support to treat pulpal exposures caused by dental trauma injuries or caries lesions with vital pulp therapies (VPTs) [1,2,3,4]. According to the recently published guidelines for dental trauma management, every effort should be made to preserve the vitality of this tissue in immature and mature teeth, recommending conservative pulpal therapy approaches [5]. Meanwhile, the European Society of Endodontology has opened the door for this paradigm shift, also recommending VPTs such as pulp capping and pulpotomy (partial and full) for cariously exposed pulps [6].

VPT aims to remove the microbial irritation and protect the exposed tissues from external stimuli by placing a well-sealing dental material. Ideally, this material should not be toxic to the pulp cells, but it should also be bioactive towards the tissues by stimulating migration, proliferation and odontogenic differentiation of the cells. Traditionally, materials based on calcium hydroxide or calcium silicate have been used for this purpose [7,8,9]. Calcium hydroxide, due to its alkaline pH, exhibits an antibacterial effect and induces superficial necrosis in the pulpal tissues. This is thought to promote odontoblast differentiation and the formation of a dentin bridge [9,10]. However, its main drawbacks are its dissolution over time, its lack of bonding to the dentin, leading to susceptibility to leakage, and the tunnel defects in the dentin bridge formed [7,11,12]. In the 1990s, mineral trioxide aggregate (MTA) appeared, a calcium silicate-based cement (CSC) commercially available as ProRoot MTA (Tulsa Dental Products, Tulsa, OK, USA), composed mainly of Portland cement and bismuth oxide [13,14]. MTA has proven excellent biocompatibility, with the ability to induce mineralization, showing high clinical success rates for VPT, generally inducing bridge formation [7,11,15,16]. However, its long setting time [17], tooth discoloration [18], high cost and difficult handling characteristics are considered its main disadvantages [8,11]. Subsequently, several other CSCs have been developed, which, depending on the purpose, can be classified into restorative cements used in VPT (Biodentine^TM^, MTA Angelus, RetroMTA and TheraCal LC) and endodontic sealers (BioRoot RCS) [8]. Biodentine^TM^ (Septodont, St. Maur-des-Fossés, France) has been manufactured to overcome the disadvantages of MTA [19], exhibiting reduced setting time [20], enhanced handling and mechanical properties [21] and adequate radiopacity [22]. It has also shown good clinical success in VPT, inducing complete dentinal bridge formation in exposed pulps of asymptomatic and symptomatic vital permanent teeth [8,23,24,25,26]. Moreover, evidence from in vitro and animal studies showed the capacity of Biodentine to promote greater mineralized tissue deposition than other CSCs [27,28]

Since the sealing capability of materials used in VPT is key, materials that bond to dentin, stimulate odontogenic differentiation of pulpal cells and induce rapid formation of a dentin bridge are ideal to allow a reliable sealing. For this purpose, bioceramic-based materials have mainly been explored, including traditional bioceramics such as hydroxyapatite [29], calcium phosphate [30], gelatine/hydroxyapatite/tricalcium phosphate composites [31] or novel sol–gel SiO_2_/ZrO_2_ ceramic composites [32]. In addition, bioactive glass (BG) is a bioceramic that exhibits advanced bioactive properties for VPT applications. The original BG, developed by Hench, is composed of 46.1 mol.% SiO_2_, 24.4 mol.% Na_2_O, 26.9 mol.% CaO and 2.6 mol.% P_2_O_5_, and it is known as 45S5 and Bioglass [33]. BG was initially used for medical applications due to its ability to bond to bone through the formation of an apatite layer on its surface [34,35]. However, it has also since been used in dentistry, incorporated into toothpaste for enamel remineralization and used for the treatment of dentin hypersensitivity [33]. In addition, BG incorporation into other dental materials, such as endodontic sealers [36,37], resin adhesives [38,39] and resin composites [40,41,42,43], has been explored. This was the topic of a recent critical review, which concludes that the addition of BG into dental composites is promising, presenting multiple benefits, especially its capacity to promote the precipitation of apatite [44]. When incorporated to an endodontic sealer, it has demonstrated in vitro capacity to promote differentiation of human periodontal ligament stem cells into cementoblast-like cells, enhancing the expression of genes related to the production of mineralized tissues [36]. Additionally, when this material was tested in a subcutaneous implantation model, it evidenced an adequate tissue reaction [37]. BG incorporated to resin adhesives showed the ability to bond and remineralize dentin [38]. It also promoted the precipitation of hydroxyapatite and calcium carbonate, improving the hybrid layer stability [39]. Similarly, experimental resin composites with BG have demonstrated multiple advantages, including the remineralization of adjacent demineralized dentin [40], acid-neutralizing properties [45], a local antimicrobial effect [41], reduction of biofilm penetration into marginal gaps [42] and enhancement of their mechanical properties [46,47].

The use of nanoparticles of BG (nBGs) with a high surface-to-volume ratio is of tremendous interest because of their larger specific surface area and enhanced bioactivity compared to the micrometric-sized particles of BG [48,49]. In addition, nBGs exhibit a higher remineralization rate of dentin [50] and an antimicrobial effect [51] when compared to microsized BG. Furthermore, resin composites containing nBGs promote the formation of a more uniform apatite layer and improve their capability to increase pH when compared to resin composites containing microsized BG [52]. nBGs form apatite in contact with the physiological fluids and they have been proven capable of inducing differentiation into a mineralizing lineage of stem cells [49,53,54]. In rat dental pulp stem cells, they increase the expression of odontogenic-related genes and the capacity for mineralization [54], and in hDPSC, they increase ALP activity, osteocalcin (OC) and dentin sialophosphoprotein (DSPP) production, and the formation of mineralized nodules [53].

The specific use of BG in VPT has been explored in animal models, showing the formation of reparative dentin and only mild inflammatory response in pulp capping procedures [55,56,57]. More recently, in a clinical trial on primary teeth, it demonstrated the formation of a dentin bridge [58]. However, there are no commercially available materials with BG currently, nor with nBGs for VPT application. For more practical reasons, this material in its powder form is not convenient to be applied as a VPT material. The materials for VPT should have good handling properties to allow the correct placement in the constricted space where pulpal exposure may occur [8]. Therefore, in general, materials used for this application are applied when recently mixed, before setting, to later achieve higher mechanical properties [8,59].

The incorporation of nBGs into BD (nBG/BD) is a possible new nanocomposite material that could integrate the handling properties and the ability to set of BD, together with its mechanical, biocompatible and bioactive characteristics, with further increased bioactivity provided by the incorporation of the nBGs. It has been previously shown that this nanocomposite presents enhanced bioactive properties in simulated body fluid, allowing a faster deposition of apatite on the surface of the material [60]. However, its ability to sustain human dental pulp stem cell (hDPSC) viability and differentiation remains largely unknown. The cellular response to the material is particularly relevant, especially its ability to induce cellular differentiation into a mineralizing lineage, since the formation of dentin in the injured area is clinically desirable. In this context, the development of this bioactive material, for dentin–pulp complex regeneration, is an interesting perspective. Therefore, the aims of this work are:

- to assess the cytocompatibility, in terms of viability, adhesion and morphology of hDPSCs, on direct contact with nBG/BD.
- to assess the ability of nBG/BD to stimulate the differentiation of hDPSCs into a mineralizing lineage.

The null hypothesis was that there would be no difference in cytocompatibility and ability to stimulate differentiation of hDPSCs into a mineralizing lineage between nBG/BD nanocomposites and BD.

## 2. Materials and Methods

### 2.1. Bioactive Glass Nanoparticle Synthesis and Nanocomposite Preparation

nBG particles (size ca. 40–70 nm) were synthesized by the sol–gel method, using the following molar composition: 58SiO_2_:40CaO:5P_2_O_5_ [61]. Briefly, a calcium-based solution was prepared by dissolving appropriate amounts of Ca(NO_3_)_2_⋅4H_2_O (Merck, Darmstadt, Germany) in 120 mL of distilled water at room temperature. A second solution was prepared by diluting tetraethylorthosilicate (TEOS 98%; Sigma, Saint Louis, MO, USA) in 60 mL of ethanol, which was added to the calcium nitrate solution, and the pH of the resulting solution was adjusted to 2.0 with nitric acid. This transparent solution was slowly dropped under vigorous stirring into a solution of NH_4_H_2_PO_4_ (May & Baker, Dagenham, England) in 1200 mL of distilled water. During the dripping process, the pH was kept at around 10 with aqueous ammonia. The reaction mixture was subjected to constant stirring for 48 h at 60 °C and allowed to stand for 24 h at room temperature. In this way, a precipitate was obtained, which was separated by centrifugation for 20 min at 12,000 rpm. This precipitate was washed through 3 cycles of centrifugation and redispersion of 40 min each. The solid obtained was frozen at −80 °C for 12 h, then lyophilized for 48 h and finally calcined at 700 °C for 3 h, producing a fine white powder of nBGs.

Nanocomposites based on nBG nanoparticles combined with BD (Septodont, Saint Maur des Fosses, France) were prepared. The 2% nBG/BD and 5% nBG/BD nanocomposites were obtained by adding 15 and 39 mg of nBG powder to the amount of BD existing in the commercial capsule, respectively. The resulting nBG/BD powders were then dry mixed within the BD capsule by using an amalgamator (Ultramat 2, SDI, Bayswater, Victoria, Australia) for 30 s. Five drops of BD liquid phase were then added to the capsule before mixing, according to the BD manufacturer’s instructions. Neat BD and GIC (Fuji II, GC America Inc, Alsip, IL, USA) were used as reference materials and they were prepared following the manufacturer’s instructions. Discs of the materials, measuring 6 mm in diameter and 1.5 mm thick, were prepared and allowed to fully set during incubation at 37 °C and 100% humidity for 24 h.

### 2.2. Nanocomposite Characterization

Discs of the nanocomposite materials (2% and 5% nBG/BD), BD and GIC were dehydrated, mounted on aluminum stubs and gold coated. Specimens were examined using scanning electron microscopy (SEM) in a JSM-IT300LV microscope (JEOL USA Inc., Peabody, MA, USA) equipped with an energy dispersive X-ray detector (EDX) and Aztec EDS software (Oxford Instruments, Abingdon, UK) for elemental analysis. SEM representative images at 1000× were obtained and EDX analysis of the surfaces of the materials was performed.

### 2.3. hDPSC Culture

The use of human cells in this study was approved by the Ethics Committee of the Faculty of Dentistry, University of Chile (Approval number PRI-ODO2018/06). Human dental pulp stem cells (hDPSCs) were isolated from human third molars, which were extracted for orthodontic reasons at the Dental Clinic, University of Chile. The extraction protocol was described by Covarrubias et al. [62].

### 2.4. hDPSC Viability Assays

hDPSCs were seeded directly onto the surface of 2% and 5% nBG/BD, BD and GIC discs (5 × 104 cells), placed in a single well of a 24-well cell culture plate and cultured in Dulbecco’s modified Eagle medium (alpha-MEM; Invitrogen, Carlsbad, CA, USA) containing 10% fetal bovine serum (FBS, Gibco, Grand Island, NY, USA), 10 mM HEPES (Gibco, Grand Island, NY, USA), 100 U/mL penicillin and 100 mg/mL streptomycin (Sigma, Saint Louis, MO, USA). Cell viability was determined at 3, 7 and 14 days of incubation by using the CellTiter 96^®^ Aqueous One Solution Cell Proliferation Assay (Promega, Madison, WI, USA), which measures the reduction of [3-(4,5-dimethylthiazol-2-yl)-5-(3-carboxymethoxyphenyl)-2-(4-sulfophenyl)-2H–tetrazolium] (MTS) to formazan by mitochondria in viable cells. Samples were incubated at 37 °C in a humidified 5% CO_2_ atmosphere. The amount of soluble formazan produced by cellular reduction of MTS was measured by a microplate reader (InfiniteM200, Tecan, Crailsheim, Germany) at a wavelength of 490 nm.

### 2.5. hDPSC Morphology and Attachment

hDPSCs were directly seeded onto the material surfaces. After 7 and 14 days of incubation, the discs with cells were fixed (2% glutaraldehyde, Sigma-Aldrich, Saint Louis, MO, USA) and stored at 4 °C before starting the dehydration process. Discs were then immersed in increasing ethanol solutions. Critical point drying of specimens using CO_2_ in an Autosamdri-815, Series A (Tousimis, Rockville, MD, USA) was performed. Samples were sputter-coated with 200 Å of gold and observed under SEM (Jeol JSM-IT300LV, JEOL USA Inc, Peabody, MA, USA). Representative micrographs of the surface of the materials after 7 and 14 days of culture were captured at 100× and 1000×.

### 2.6. Alkaline Phosphate Activity of hDPSCs

The capacity of the dental materials to stimulate the differentiation of hDPSCs into a mineralizing lineage was assessed by measuring the activity of the alkaline phosphatase (ALP) enzyme. ALP activity of hDPSCs cultured with the discs and a control (CT, without discs) was determined after 7 and 14 days of culture by a colorimetric dephosphorylation assay of a p-nitrophenyl phosphate reagent (Sigma, Saint Louis, MO, USA), which was followed by the measuring of the absorbance with a microplate reader (InfiniteM200, Tecan, Crailsheim, Germany) at a wavelength of 405 nm.

### 2.7. Statistical Analysis

Data obtained from the viability and alkaline phosphate activity assays were evaluated using SPSS software (SPSS Inc., Chicago, IL, USA). The results obtained for all materials were submitted to the Shapiro–Wilk normality test. After proving the normality of the sample data distribution, the data were submitted to a one- and two-way ANOVA and post hoc Tukey test at a 5% level of significance.

## 3. Results

### 3.1. Nanocomposite Characterization

**Figure 1:**
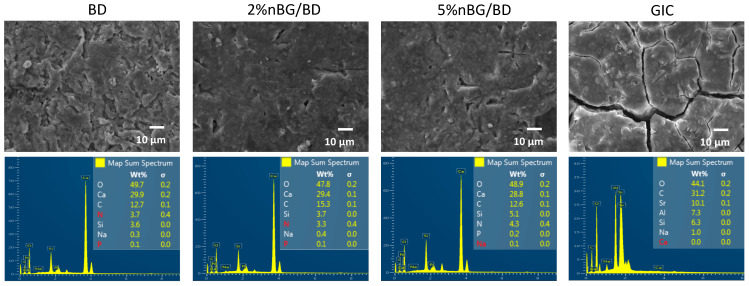
Representative SEM images (accelerating voltage of 20 kV, working distance of 10.3–10.6 mm, magnification of 1000×) and EDX elemental analysis of BD, 2% nBG/BD, 5% nBG/BD and GIC set cement surfaces.

SEM images and EDX elemental analysis of the surface of the set cements are presented in Figure 1. The surfaces of the nanocomposite discs (2% and 5% nBG/BD) appear similar to unmodified BD. BD, 2% nBG/BD and 5% nBG/BD are mainly composed (>10% by weight) of oxygen, calcium and carbon and a smaller amount (1–10% by weight) of silica and nitrogen, with traces (<1% by weight) of sodium and phosphorous. In contrast, the GIC surface presents visible cracks and it is composed mainly of oxygen, carbon and strontium and a smaller amount of aluminum and silica, with traces of sodium.

### 3.2. hDPSC Viability

**Figure 2:**
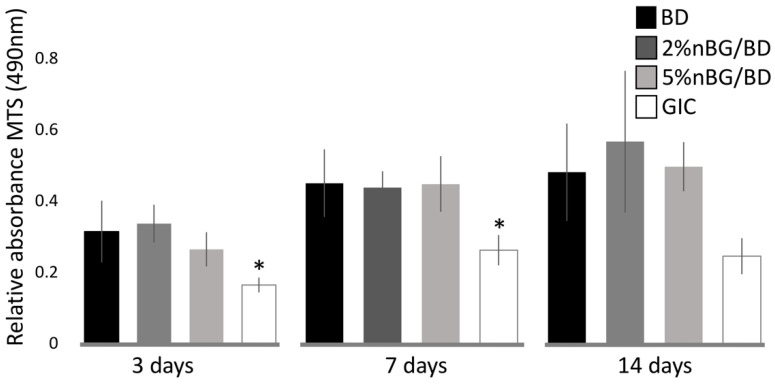
Cell viability of hDPSCSs cultured with BD, 2% nBG/BD, 5% nBG/BD and GIC at different culture times as determined by the MTS assay. Values are combined from 2 experiments (*n* = 4/experiment), standard deviations are represented by vertical bars. *: Statistically significant difference compared with BD (*p* < 0.05).

MTS cell viability of hDPSCs cultured in direct contact with the materials was assessed after 3, 7 and 14 days of incubation (Figure 2). The viability of hDPSCs cultured on 2% and 5% nBG/BD did not present statistical differences in comparison with those grown on unmodified BD. However, the viability of hDPCSs cultured with GIC was significantly lower than when cultured on BD and nanocomposites.

### 3.3. hDPSC Morphology and Adhesion

**Figure 3:**
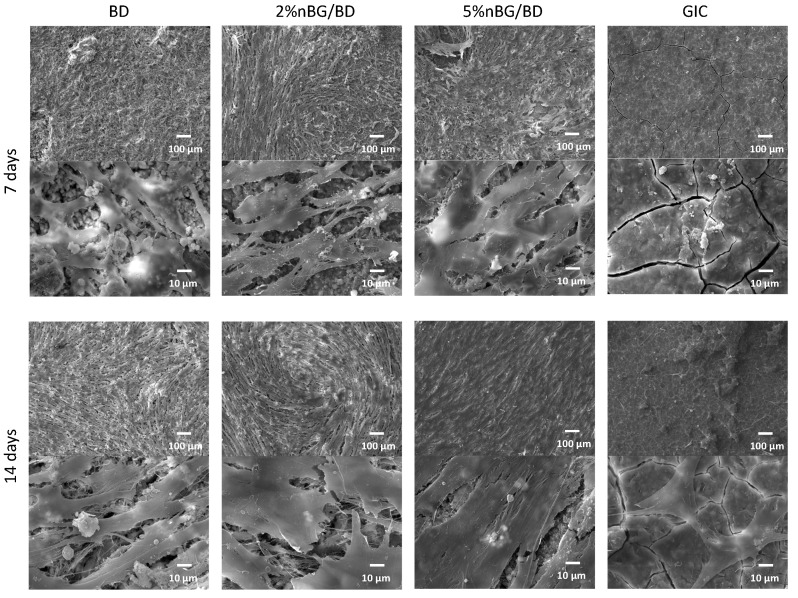
Representative SEM images (accelerating voltage of 20 kV, working distance of 10.0–11.4 mm, magnification 100× and 1000×) of hDPSCs cultured with BD, 2% nBG/BD, 5% nBG/BD and GIC after 7 and 14 days of incubation.

SEM images of hDPSCs attached to the cement surfaces are shown in Figure 3. After 7 days, cells covered almost the entire surface of the BD and nBG/BD materials, exhibiting a flattened morphology with cytoplasmic extensions projecting from the cells to adjacent cells. After 14 days of incubation, an increased density of cells and a thicker cell layer were observed. White and globular areas with the appearance of mineralized nodules could be also noted. In GIC samples, after 7 days of incubation, scarce cells with low adhesion were observed, and after 14 days, cells appeared more flattened and adhered although with notably lower cell density when compared to the other materials.

### 3.4. ALP Activity of hDPSCs

**Figure 4:**
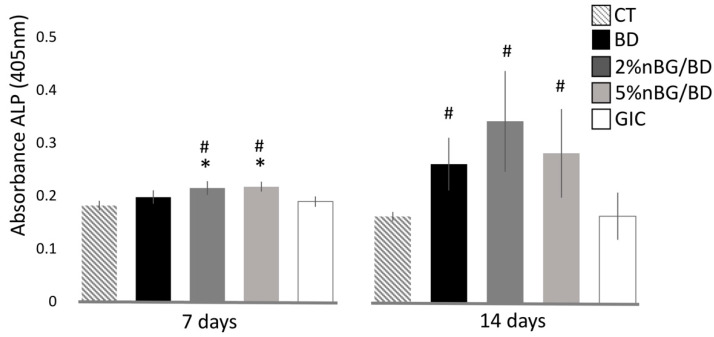
ALP activity of hDPSCs cultured with BD, 2% nBG/BD, 5% nBG/BD and GIC at different times. Values are means combined from 2 experiments (*n* = 4/experiment), standard deviations are represented by vertical bars. #: Statistically significant difference compared with control (*p* < 0.05). *: Statistically significant difference compared with BD (*p* < 0.05).

The ability of the nanocomposites to stimulate differentiation of hDPSCs towards a mineralizing lineage was assessed by quantifying the expression of ALP enzyme (Figure 4). Statistically, significantly higher ALP activity values were measured in hDPSCs in contact with 2 and 5% nBG/BD after 7 days of culture, when compared to hDPSCs in contact with the control (cells without material), BD and GIC. After 14 days of culture, the nanocomposites and BD presented a significantly higher ALP activity when compared to CT and GIC.

## 4. Discussion

In the present study, the incorporation of nBGs to a calcium silicate cement improved its ability to induce odontogenic differentiation, without generating significant changes in the adhesion and cell viability of hDPSCs.

The cytocompatibility of the nanocomposite materials was evaluated with the materials in direct contact with the hDPSC culture since this is the relationship that is established in VPTs. Cell viability measured through MTS mitochondrial activity and cell adhesion observations showed that the incorporation of nBGs in contents of 2 and 5% to BD does not affect the viability nor the adhesion of hDPSCs. To our best knowledge, no studies about cytocompatibility of nBG/BD composites have been reported. However, the viability of hDPSCs in contact with nBG-modified composites has been studied by means of different vehicles such as polymer hydrogels [63], synthetic polymers [64] and chitosan scaffolds [65]. In these studies, similarly to that observed in the current work, the presence of BG did not affect the adhesion and proliferation of the cells, with a cytocompatibility similar to the controls without BG.

SEM analysis of cells adhered to the materials revealed that hDPSCs behaved similarly when in contact with nBG/BD nanocomposites and BD. The cells were flattened, forming a well-organized layer covering the entire surface, with multiple extensions between the cells and towards the surface of the materials, indicating an effective cellular adhesion. In addition, the presence of nodules with a mineralizing appearance was also observed [62], which could be coupled to the differentiation process of the cells towards a mineralizing lineage. In contrast, the surface of the GIC was smooth and cracked, with poorly adhered cells, isolated and with a contracted appearance [66].

Cell differentiation into a mineralizing lineage was confirmed by determining the activity of ALP, an enzyme involved in the mineralizing process. hDPSCs cultured on the nBG/BD nanocomposites showed an early induction of ALP production after 7 days of culture, statistically higher than those incubated with neat BD. After 14 days, cells on nBG/BD and BD showed increased ALP activity compared to those cultured with control and GIC. Especially, BD loaded with 2% nBG induced the highest ALP activity, indicating that this nanoparticle content favors the stimulation of the cell differentiation process towards a mineralizing lineage. These results can be explained by the demonstrated capacity of nBGs to induce both osteogenic [34,67] and odontogenic differentiation [68,69,70]. nBGs have shown their capacity to promote the migration, adhesion and expression of odontogenic-related proteins and genes in hDPSCs, which has been mainly attributed to the release of Si and Ca ions [53,68,70]. In contrast with microsized particles, nBGs generate a faster release of soluble ions, which are capable of chemically driving hDPSCs along a mineralization pathway [68]. In addition, the application of microsized BG in a pulp capping procedure in primary human teeth has been shown to promote dentin bridge formation [58].

hDPSCs were used in this study to evaluate the cytocompatibility and bioactivity of the nanocomposite developed, since they are widely used in in vitro studies for the evaluation of dental materials. Their isolation is not invasive (using extracted semi-included third molars) and allows the creation of an approximation of how the cells of the dental pulp will behave when they come into direct contact with the dental material and its components [71]. One of the relevant characteristics of hDPSCs is their differentiation potential, which can be into odontoblasts, osteocytes/osteoblasts, adipocytes, chondrocytes and neural cells [72]. This capacity for differentiation allows the dental pulp to form dentin in response to a stimulus such as dental caries or trauma injuries. However, for this to occur, the vitality of the dental pulp must be preserved, hence the importance of evaluating cytocompatibility in materials for VPT [73].

Regarding the limitations of this study, additional studies are necessary to establish the safety and efficacy of the use of this material for clinical application. The results provide information about the in vitro cellular responses and whether these responses will be replicated in clinical conditions remains unknown. In addition, although the ALP activity of hDPSCs was analyzed to explore possible cell differentiation into a mineralizing type, the gene expression related to the formation of dentin was not studied. During dentinogenesis, several other genes, proteins and markers are detected, such as DSPP, dentin sialoprotein (DSP), dentin phosphoprotein (DPP), dentine matrix protein-1 (DMP-1) and OC, whose expression would be relevant to study [73]. nBGs have been shown to increase the expression of odontogenic genes, osteocalcin and DSPP protein production in hDPSCs [49]. Therefore, it would be interesting to know if this effect is maintained when they are incorporated into BD. Further investigations are also necessary to study the inflammatory response to this material, since inflammation and regeneration are of particular significance within the non-extensive space in the dental pulp tissue [69,74]. It would also be relevant to perform future research using animal models, which could provide histological evidence of hard tissue deposition and sealing ability, before clinical testing [75,76].

Within the limitations of this in vitro study, the results indicate that this nanocomposite could be a promising material for use in direct contact with the injured dentin–pulp complex, which could lead to faster repair. These outcomes raise the interesting possibility of using the nanocomposite in direct contact with dental pulp tissues to contribute to the preservation of natural dental tissue.

## 5. Conclusions

The incorporation of nBGs in a calcium silicate cement does not alter the cytocompatibility, in terms of viability, adhesion and morphology of hDPSCs, compared to the neat cement.

The nBG/BD nanocomposite exhibited a higher capacity to stimulate the differentiation of hDPSCs into a mineralizing lineage than BD.

The cellular properties of the nBG/BD nanocomposite make it a promising material to be used in VPT, which could lead to faster dentin formation and therefore to the healing and repair of the dentin–pulp complex.

## Data Availability

The data presented in this study are available on request from the corresponding author.

## References

[1] Aguilar P., Linsuwanont P. (2011). Vital pulp therapy in vital permanent teeth with cariously exposed pulp: A systematic re-view. J. Endod..

[2] Elmsmari F., Ruiz X.-F., Miró Q., Feijoo-Pato N., Durán-Sindreu F., Olivieri J.G. (2019). Outcome of Partial Pulpotomy in Cariously Exposed Posterior Permanent Teeth: A Systematic Review and Meta-analysis. J. Endod..

[3] Wang G., Wang C., Qin M. (2017). Pulp prognosis following conservative pulp treatment in teeth with complicated crown fractures-A retrospective study. Dent. Traumatol..

[4] Bimstein E., Rotstein I. (2016). Cvek pulpotomy—Revisited. Dent. Traumatol..

[5] Bourguignon C., Cohenca N., Lauridsen E., Flores M.T., O’Connell A.C., Day P.F., Tsilingaridis G., Abbott P.V., Fouad A.F., Hicks L. (2020). International Association of Dental Traumatology guidelines for the management of traumatic dental injuries: 1. Fractures and luxations. Dent. Traumatol..

[6] Duncan H.F., Galler K.M., Tomson P.L., Simon S., El-Karim I., Kundzina R., Krastl G., Dammaschke T., Fransson H., Markvart M. (2019). European Society of Endodontology position statement: Management of deep caries and the exposed pulp. Int. Endod. J..

[7] Bakland L.K., Andreasen J.O. (2011). Will mineral trioxide aggregate replace calcium hydroxide in treating pulpal and periodontal healing complications subsequent to dental trauma? A review. Dent. Traumatol..

[8] Kunert M., Lukomska-Szymanska M. (2020). Bio-Inductive Materials in Direct and Indirect Pulp Capping—A Review Article. Mater..

[9] Komabayashi T., Zhu Q., Eberhart R., Imai Y. (2016). Current status of direct pulp-capping materials for permanent teeth. Dent. Mater. J..

[10] Schwendicke F., Brouwer F., Schwendicke A., Paris S. (2016). Different materials for direct pulp capping: Systematic review and meta-analysis and trial sequential analysis. Clin. Oral Investig..

[11] Parirokh M., Torabinejad M., Dummer P.M.H. (2017). Mineral trioxide aggregate and other bioactive endodontic cements: An updated overview—part I: Vital pulp therapy. Int. Endod. J..

[12] Asgary S., Eghbal M.J., Parirokh M., Ghanavati F., Rahimi H. (2008). A comparative study of histologic response to different pulp capping materials and a novel endodontic cement. Oral Surg. Oral Med. Oral Pathol. Oral Radiol. Endodontol..

[13] Camilleri J., Montesin F.E., Brady K., Sweeney R., Curtis R.V., Ford T.R.P. (2005). The constitution of mineral trioxide aggregate. Dent. Mater..

[14] Torabinejad M., White D. (1998). Tooth Filling Material and Use. U.S. Patent.

[15] Matsuura T., Ziauddin S.M., Kawata-Matsuura V.K.S., Sugimoto K., Yamada S., Yoshimura A. (2021). Long-term clinical and radiographic evaluation of the effectiveness of direct pulp capping materials: A meta-analysis. Dent. Mater. J..

[16] Cushley S., Duncan H.F., Lappin M.J., Chua P., Elamin A.D., Clarke M., El-Karim I.A. (2020). Efficacy of direct pulp cap-ping for management of cariously exposed pulps in permanent teeth: A systematic review and meta-analysis. Int. Endod. J..

[17] Asgary S., Shahabi S., Jafarzadeh T., Amini S., Kheirieh S. (2008). The Properties of a New Endodontic Material. J. Endod..

[18] Yun D.-A., Park S.-J., Lee S.-R., Min K.-S. (2015). Tooth discoloration induced by calcium-silicate-based pulp-capping materials. Eur. J. Dent..

[19] Bahabri R., Krsoum M. (2020). Biodentine: Perforation, retrograde filling, and vital pulp therapy. A review. Int. J. Med. Dent..

[20] Grech L., Mallia B., Camilleri J. (2013). Investigation of the physical properties of tricalcium silicate cement-based root-end filling materials. Dent. Mater..

[21] Nielsen M.J., Casey J.A., VanderWeele R.A., Vandewalle K.S. (2016). Mechanical properties of new dental pulp-capping materials. Gen. Dent..

[22] Corral C., Negrete P., Estay J., Osorio S., Covarrubias C., Junior O.B.D.O., Barud H. (2018). Radiopacity and Chemical Assessment of New Commercial Calcium Silicate-Based Cements. Int. J. Odontostomatol..

[23] Brizuela C., Ormeño A., Cabrera C., Cabezas R., Silva C.I., Ramírez V., Mercade M. (2017). Direct Pulp Capping with Calcium Hydroxide, Mineral Trioxide Aggregate, and Biodentine in Permanent Young Teeth with Caries: A Randomized Clinical Trial. J. Endod..

[24] Nowicka A., Lipski M., Parafiniuk M., Sporniak-Tutak K., Lichota D., Kosierkiewicz A., Kaczmarek W., Buczkowska-Radlińska J. (2013). Response of Human Dental Pulp Capped with Biodentine and Mineral Trioxide Aggregate. J. Endod..

[25] Katge F.A., Patil D.P. (2017). Comparative Analysis of 2 Calcium Silicate–based Cements (Biodentine and Mineral Trioxide Aggregate) as Direct Pulp-capping Agent in Young Permanent Molars: A Split Mouth Study. J. Endod..

[26] Harms C.S., Schäfer E., Dammaschke T. (2019). Clinical evaluation of direct pulp capping using a calcium silicate cement—treatment outcomes over an average period of 2.3 years. Clin. Oral Investig..

[27] Sequeira D.B., Oliveira A.R., Seabra C.M., Palma P.J., Ramos C., Figueiredo M.H., Santos A.C., Cardoso A.L., Peça J., Santos J.M. (2021). Regeneration of pulp-dentin complex using human stem cells of the apical papilla: In vivo interaction with two bioactive materials. Clin. Oral Investig..

[28] Luo Z., Kohli M.R., Yu Q., Kim S., Qu T., He W.-X. (2014). Biodentine Induces Human Dental Pulp Stem Cell Differentiation through Mitogen-activated Protein Kinase and Calcium-/Calmodulin-dependent Protein Kinase II Pathways. J. Endod..

[29] Hanafy A.K., Shinaishin S.F., Eldeen G.N., Aly R.M. (2018). Nano Hydroxyapatite & Mineral Trioxide Aggregate Efficiently Promote Odontogenic Differentiation of Dental Pulp Stem Cells. Open Access Maced. J. Med. Sci..

[30] Nam S., Won J.-E., Kim C.-H., Kim H.-W. (2011). Odontogenic Differentiation of Human Dental Pulp Stem Cells Stimulated by the Calcium Phosphate Porous Granules. J. Tissue Eng..

[31] Gu Y., Bai Y., Zhang D. (2018). Osteogenic stimulation of human dental pulp stem cells with a novel gelatin-hydroxyapatite-tricalcium phosphate scaffold. J. Biomed. Mater. Res. Part A.

[32] Catauro M., Barrino F., Poggetto G.D., Milazzo M., Blanco I., Ciprioti S.V. (2020). Structure, drug absorption, bioactive and antibacterial properties of sol-gel SiO2/ZrO2 materials. Ceram. Int..

[33] Jones J.R. (2013). Review of bioactive glass: From Hench to hybrids. Acta Biomater..

[34] Hench L.L. (2006). The story of Bioglass^®^. J. Mater. Sci. Mater. Med..

[35] Hench L.L. (1991). Bioceramics: From Concept to Clinic. J. Am. Ceram. Soc..

[36] Rodríguez-Lozano F., Collado-González M., Tomás-Catalá C., García-Bernal D., López S., Oñate-Sánchez R., Moraleda J., Murcia L. (2019). GuttaFlow Bioseal promotes spontaneous differentiation of human periodontal ligament stem cells into cementoblast-like cells. Dent. Mater..

[37] Santos J.M., Pereira S., Sequeira D.B., Messias A.L., Martins J.B., Cunha H., Palma P.J., Santos A.C. (2019). Biocompatibility of a bioceramic silicone-based sealer in subcutaneous tissue. J. Oral Sci..

[38] Abuna G., Campos P., Hirashi N., Giannini M., Nikaido T., Tagami J., Sinhoreti M.A.C., Geraldeli S. (2021). The ability of a nanobioglass-doped self-etching adhesive to re-mineralize and bond to artificially demineralized dentin. Dent. Mater..

[39] Carvalho E.M., Ferreira P.V.C., Gutiérrez M.F., Sampaio R.F., Carvalho C.N., de Menezes A.S., Loguercio A.D., Bauer J. (2021). Development and characterization of self-etching adhesives doped with 45S5 and niobophosphate bioactive glasses: Physicochemical, mechanical, bioactivity and interface properties. Dent. Mater..

[40] Jang J.-H., Lee M.G., Ferracane J.L., Davis H., Bae H.E., Choi N., Kim D.-S. (2018). Effect of bioactive glass-containing resin composite on dentin remineralization. J. Dent..

[41] Korkut E., Torlak E., Altunsoy M. (2016). Antimicrobial and mechanical properties of dental resin composite containing bioactive glass. J. Appl. Biomater. Funct. Mater..

[42] Khvostenko D., Hilton T., Ferracane J., Mitchell J., Kruzic J. (2016). Bioactive glass fillers reduce bacterial penetration into marginal gaps for composite restorations. Dent. Mater..

[43] Par M., Spanovic N., Tauböck T.T., Attin T., Tarle Z. (2019). Degree of conversion of experimental resin composites containing bioactive glass 45S5: The effect of post-cure heating. Sci. Rep..

[44] Tiskaya M., Shahid S., Gillam D., Hill R. (2021). The use of bioactive glass (BAG) in dental composites: A critical review. Dent. Mater..

[45] Yang S.-Y., Piao Y.-Z., Kim S.-M., Lee Y.-K., Kim K.-N., Kim K.-M. (2013). Acid neutralizing, mechanical and physical properties of pit and fissure sealants containing melt-derived 45S5 bioactive glass. Dent. Mater..

[46] Khvostenko D., Mitchell J.C., Hilton T.J., Ferracane J.L., Kruzic J.J. (2013). Mechanical performance of novel bioactive glass containing dental restorative composites. Dent. Mater..

[47] Khodaei M., Nejatidanesh F., Shirani M.J., Valanezhad A., Watanabe I., Savabi O. (2019). The effect of the nano- bioglass reinforcement on magnesium based composite. J. Mech. Behav. Biomed. Mater..

[48] Misra S.K., Mohn D., Brunner T.J., Stark W.J., Philip S.E., Roy I., Salih V., Knowles J.C., Boccaccini A.R. (2008). Comparison of nanoscale and microscale bioactive glass on the properties of P(3HB)/Bioglass^®^ composites. Biomaterials.

[49] Ajita J., Saravanan S., Selvamurugan N. (2015). Effect of size of bioactive glass nanoparticles on mesenchymal stem cell proliferation for dental and orthopedic applications. Mater. Sci. Eng. C.

[50] Vollenweider M., Brunner T.J., Knecht S., Grass R.N., Zehnder M., Imfeld T., Stark W.J. (2007). Remineralization of human dentin using ultrafine bioactive glass particles. Acta Biomater..

[51] Waltimo T., Brunner T., Vollenweider M., Stark W., Zehnder M. (2007). Antimicrobial Effect of Nanometric Bioactive Glass 45S5. J. Dent. Res..

[52] Odermatt R., Par M., Mohn D., Wiedemeier D.B., Attin T., Tauböck T.T. (2020). Bioactivity and Physico-Chemical Properties of Dental Composites Functionalized with Nano- vs. Micro-Sized Bioactive Glass. J. Clin. Med..

[53] Gong W., Huang Z., Dong Y., Gan Y., Li S., Gao X., Chen X. (2014). Ionic Extraction of a Novel Nano-sized Bioactive Glass Enhances Differentiation and Mineralization of Human Dental Pulp Cells. J. Endod..

[54] Lee J.-H., Kang M.-S., Mahapatra C., Kim H.-W. (2016). Effect of Aminated Mesoporous Bioactive Glass Nanoparticles on the Differentiation of Dental Pulp Stem Cells. PLoS ONE.

[55] Oguntebi B., Clark A., Wilson J. (1993). Pulp Capping with Bioglass^®^ and Autologous Demineralized Dentin in Miniature Swine. J. Dent. Res..

[56] Long Y., Liu S., Zhu L., Liang Q., Chen X., Dong Y. (2017). Evaluation of Pulp Response to Novel Bioactive Glass Pulp Capping Materials. J. Endod..

[57] Zhu N., Chatzistavrou X., Papagerakis P., Ge L., Qin M., Wang Y. (2019). Silver-Doped Bioactive Glass/Chitosan Hydrogel with Potential Application in Dental Pulp Repair. ACS Biomater. Sci. Eng..

[58] Ahmadvand M., Haghgoo R. (2016). Evaluation of pulpal response of deciduous teeth after direct pulp capping with bioactive glass and mineral trioxide aggregate. Contemp. Clin. Dent..

[59] Hanna S.N., Alfayate R.P., Prichard J. (2020). Vital pulp therapy an insight over the available literature and future expectations. Eur. Endod. J..

[60] Nuñez C.C., Covarrubias C., Fernandez E., De Oliveira O.B. (2017). Enhanced bioactive properties of BiodentineTM modified with bioactive glass nanoparticles. J. Appl. Oral Sci..

[61] Valenzuela F., Covarrubias C., Martínez C., Smith P., Díaz-Dosque M., Yazdani-Pedram M. (2012). Preparation and bioactive properties of novel bone-repair bionanocomposites based on hydroxyapatite and bioactive glass nanoparticles. J. Biomed. Mater. Res. Part B Appl. Biomater..

[62] Covarrubias C., Agüero A., Maureira M., Morelli E., Escobar G., Cuadra F., Peñafiel C., Von Marttens A. (2019). In situ preparation and osteogenic properties of bionanocomposite scaffolds based on aliphatic polyurethane and bioactive glass nanoparticles. Mater. Sci. Eng. C.

[63] Sevari S.P., Shahnazi F., Chen C., Mitchell J.C., Ansari S., Moshaverinia A. (2019). Bioactive glass-containing hydrogel delivery system for osteogenic differentiation of human dental pulp stem cells. J. Biomed. Mater. Res. Part A.

[64] Kim G.-H., Park Y.-D., Lee S.-Y., El-Fiqi A., Kim J.-J., Lee E.-J., Kim H.-W., Kim E.-C. (2014). Odontogenic stimulation of human dental pulp cells with bioactive nanocomposite fiber. J. Biomater. Appl..

[65] Zhu N., Chatzistavrou X., Ge L., Qin M., Papagerakis P., Wang Y. (2019). Biological properties of modified bioactive glass on dental pulp cells. J. Dent..

[66] Nuñez C.M.C., Bosomworth H.J., Field C., Whitworth J.M., Valentine R.A. (2014). Biodentine and Mineral Trioxide Aggregate Induce Similar Cellular Responses in a Fibroblast Cell Line. J. Endod..

[67] Wang S., Huang G., Dong Y. (2020). Directional Migration and Odontogenic Differentiation of Bone Marrow Stem Cells Induced by Dentin Coated with Nanobioactive Glass. J. Endod..

[68] Wang S., Gao X., Gong W., Zhang Z., Chen X., Dong Y. (2014). Odontogenic differentiation and dentin formation of dental pulp cells under nanobioactive glass induction. Acta Biomater..

[69] Mocquot C., Attik N., Pradelle-Plasse N., Grosgogeat B., Colon P. (2020). Bioactivity assessment of bioactive glasses for dental applications: A critical review. Dent. Mater..

[70] Wang S., Hu Q., Gao X., Dong Y. (2016). Characteristics and Effects on Dental Pulp Cells of a Polycaprolactone/Submicron Bioactive Glass Composite Scaffold. J. Endod..

[71] Morsczeck C., Reichert T.E. (2018). Dental stem cells in tooth regeneration and repair in the future. Expert Opin. Biol. Ther..

[72] Nuti N., Corallo C., Chan B.M.F., Ferrari M., Gerami-Naini B. (2016). Multipotent Differentiation of Human Dental Pulp Stem Cells: A Literature Review. Stem Cell Rev. Rep..

[73] Da Rosa W.L.O., Piva E., Da Silva A.F. (2018). Disclosing the physiology of pulp tissue for vital pulp therapy. Int. Endod. J..

[74] Giraud T., Jeanneau C., Rombouts C., Bakhtiar H., Laurent P., About I. (2019). Pulp capping materials modulate the balance between inflammation and regeneration. Dent. Mater..

[75] Song M., Kim S., Kim T., Park S., Shin K.-H., Kang M., Park N.-H., Kim R. (2017). Development of a Direct Pulp-capping Model for the Evaluation of Pulpal Wound Healing and Reparative Dentin Formation in Mice. J. Vis. Exp..

[76] Dammaschke T. (2010). Rat molar teeth as a study model for direct pulp capping research in dentistry. Lab. Anim..

